# *Ixodes scapularis *tick serine *p*roteinase inhibitor (serpin) gene family; annotation and transcriptional analysis

**DOI:** 10.1186/1471-2164-10-217

**Published:** 2009-05-12

**Authors:** Albert Mulenga, Rabuesak Khumthong, Katelyn C Chalaire

**Affiliations:** 1 Department of Entomology, Texas A&M University, 2475 TAMU, Minnie Belle Heep center, College Station, Texas 77843, USA

## Abstract

**Background:**

Serine proteinase inhibitors (Serpins) are a large superfamily of structurally related, but functionally diverse proteins that control essential proteolytic pathways in most branches of life. Given their importance in the biology of many organisms, the concept that ticks might utilize serpins to evade host defenses and immunizing against or disrupting their functions as targets for tick control is an appealing option.

**Results:**

A sequence homology search strategy has allowed us to identify at least 45 tick serpin genes in the *Ixodes scapularis *genome that are structurally segregated into 32 intronless and 13 intron-containing genes. Nine of the intron-containing serpins occur in a cluster of 11 genes that span 170 kb of DNA sequence. Based on consensus amino acid residues in the reactive center loop (RCL) and signal peptide scanning, 93% are putatively inhibitory while 82% are putatively extracellular. Among the 11 different amino acid residues that are predicted at the P1 sites, 16 sequences possess basic amino acid (R/K) residues. Temporal and spatial expression analyses revealed that 40 of the 45 serpins are differentially expressed in salivary glands (SG) and/or midguts (MG) of unfed and partially fed ticks. Ten of the 38 serpin genes were expressed from six to 24 hrs of feeding while six and fives genes each are predominantly or exclusively expressed in either MG and SG respectively.

**Conclusion:**

Given the diversity among tick species, sizes of tick serpin families are likely to be variable. However this study provides insight on the potential sizes of serpin protein families in ticks. Ticks must overcome inflammation, complement activation and blood coagulation to complete feeding. Since these pathways are regulated by serpins that have basic residues at their P1 sites, we speculate that *I. scapularis *may utilize some of the serpins reported in this study to manipulate host defense. We have discussed our data in the context of advances on the molecular physiology of *I. scapularis*. Although the paper is descriptive, this study provides the first step toward a comprehensive understanding of serpins in tick physiology.

## Background

Ticks, segregated into two families; Ixodidae (hard ticks) and Argasidae (soft ticks) are important vectors of several pathogens with a global veterinary and public health impact [1,2]. Although research on ticks has for the most part been directed towards agricultural interests, ticks have been recognized as the second most important vectors of human disease agents after mosquitoes [2]. Globally, the impact of tick borne disease agents on public health has been on a dramatic rise [3-6] since the discovery of *Borelia burdgoferi *as the causative agent for Lyme disease in 1982 [7,8]. Literature reviews by Parola and Roult [3] listed 15 new tick borne bacterial agents being discovered or recognized as human pathogens between 1982 and 2004. In the United States ticks transmit more causative agents of vector borne diseases than any other vector arthropod [9]. Tick borne human diseases reported in the USA include Babesiosis, ehrlichisosis, Southern Tick-Associated Rash illness (STARI), Lyme disease, tick-borne relapsing fever, anaplasmosis and Rocky Mountain spotted fever, [9]. From a human health perspective, *Ixodes scapularis *and its close relatives, *I. pacificus, I. ricinus *and *I. persulcatus *and *I. holocyclus *are the most important ticks as they transmit the majority of emerging human disease pathogens. The importance of these ticks to human health was the key justification for funding of the *I. scapularis *genome, sequencing project [10]. Key anticipated outcomes of the tick genome sequencing project will be provision of opportunities to identify unique tick genes that could be exploited for tick control and, thus the control of tick borne diseases [10]. We are interested in understanding the role of the serine proteinase inhibitors (serpin) in tick physiology and feeding.

Serpins represent the largest family of proteinase inhibitors that is widely conserved across taxa, from animals to plants, viruses and bacteria [11-18]. Of the 68 families of proteinase inhibitors listed on the MEROPS database (version 7.6, , [19]), the serpin family (denoted as I4) has the largest number of entries. In humans, serpins make up 2% of total blood plasma proteins [20] and are involved in the regulation of important pathways such as blood coagulation, complement activation, inflammation, fertilization and food digestion [11-14]. The importance of serpins in humans can be attested to by more than 90 human diseases such as cirrhosis, emphysema, blood coagulation disorders and dementia that arises from serpin malfunctions due to mutation [12]. In arthropods, serpins were linked to immunity in mosquitoes [21,22], the fruit fly [23-26] and the tobacco hornworm [27], development in the fruit fly [28], control of the hemolymph coagulation cascade in the horseshoe crab [29-31]. Given the importance of serpins in the biology of multicellular organisms, it has been hypothesized that, ticks might utilize serpins to evade host defenses and immunizing against or disrupting functions of these proteins is an appealing option for designing new tick control strategies [17,32].

More than 30 serpin encoding cDNAs have now been cloned in several economically and medically important ticks including *Amblyomma americanum *[33], *R. appendiculatus *[17] and *I. ricinus *[34] and *I. scapularis *[35]. Serpin encoding ESTs and cDNA sequences of *I. scapularis *and *R. microplus *cited in [33] are also available in GeneBank. Studies by Sugino et al., [36], Imamura et al., [37] and Prevot et al., [38] have provided encouraging evidence suggesting the potential of serpins as targets for tick control. These authors [36-38] showed that feeding of ticks on animals immunized with recombinant tick serpins caused ticks to obtain smaller blood meals and as consequence reduced tick fecundity and mortality. These findings clearly suggest that serpins play important roles in tick physiology.

Given the availability of *I. scapularis *genome sequence information in GeneBank and at VectorBase [38] we thought to get some insight on the size of the serpin protein family in ticks. We report here on the identification and characterization at least 45 *I. scapularis *serpin genes. A sequence based analyses show that ~93% of these serpins are putatively inhibitory with 88% (31/35) of full-length serpins being putatively extracellular. Our RT-PCR expression analyses data demonstrated that 84% (38/45) are differentially expressed in midguts and salivary glands of unfed and partially fed ticks. We have discussed our findings towards advances in *I. scapularis *tick molecular biology.

## Methods

### Discovery of *Ixodes scapularis *serpin genes

**Figure 1:**
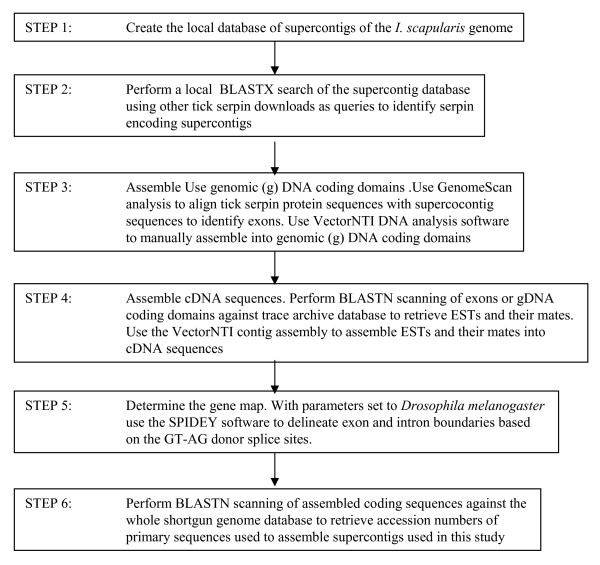
**Flow chart of the strategy used to annotate *Ixodes scapularis *serpins**. The FASTA3 version 3.4 software [41] was used to perform a local BLASTX search of the local database of supercontigs of the *I. scapularis *genome sequence data using tick serpin downloads from GeneBank as queries.

The strategy to annotate serpin genes in the *I. scapularis *genome is summarized in figure 1. In the **first step**, we downloaded supercontig DNA sequences from VectorBase [39,40] and created a local database. In the **second step**, the supercontig database was subjected to BLASTX homology search using the FASTA3 software version 3.4 [41] to identify serpin-encoding supercontigs. Queries for this search, included α_1_-antitrypsin and 30 tick serpin sequences; 17 *A. americanum *[33], one *H. longicornis *[36], four *R. appendiculatus *[17], six *I. ricinus *(DQ915842, DQ915843, DQ915844, DQ915845 and AJ269658), and two in *I. scapularis *(AAM93649 and AAV80788 [35]). In the **third step**, coding genomic DNA sequences were assembled using GenomeScan analysis [42] and the VectorNTI DNA analysis software (Invitrogen, Carlsbard, CA; version 10, academic free license). GenomeScan analysis [42] was used to align supercontig sequences with tick serpin protein sequences that were used as queries. This alignment identified exons, which were manually assembled into genomic coding sequences using the VectorNTI DNA analysis software (Invitrogen, Carlsbard, CA; version 10, academic free license). In **step four **cDNA sequences were assembled. This was done by scanning genomic DNA coding sequences from step three against the *I. scapularis *trace archive EST database. Trace archive ESTs showing e-values of zero (probability that the observation was not by chance) and their mates were retrieved and assembled using ContigExpress (Vector NTI package version 10). Prior to assembly any vector sequence contamination was removed using vector screen at NCBI. In **step five**, exon and intron boundaries were delineated based on donor-acceptor splice sites (GT-AG). This was accomplished by mapping cDNA sequences onto appropriate supercontig sequences using the Spidey software [43] at NCBI with parameters set to *Drossophila melanogaster*. In **step six**, coding domains were scanned against the whole shotgun genome database to retrieve accession numbers for primary sequences that were used to assemble the supercontigs used in this study.

### Bioinformatics analyses

For comparisons to known serpins, assembled coding sequences were scanned against known protein entries in GenBank using the BLASTx or BLASTp homology search program. To validate their accuracy, inferred amino acid sequences were inspected for two amino acid motifs, "NAVYKFG" and "DVNEEG," that are conserved in most known serpins [30,44]. Full-length and partial sequences were determined on the basis that, a typical serpin molecule ranges between 350–450 amino acid residues long [11,12]. To gain insight on probable functionality, inferred amino acid sequences were scanned against amino acid motif entries in ScanProsite and signal peptides in the SignalP servers (ExPASY Proteomics Server ). The reactive center loop (RCL) which determines the functionality (inhibitory and non-inhibitory) of a serpin molecule was determined based on consensus 20/21 residue peptide "p17 [E]-p16 [E/K/R]-p15 [G]-p14 [T/S]-p13 [X]-p12-9 [AGS]-p8-1 [X]-p1' -4' " in carboxy-terminus region [11,45]. The numbering here is based on the convention in which residues on the amino terminal side of scissile bond (p1-p1') are labeled as p17 to p1 and those on the carboxy terminal side are labeled as p1' -p4' [46]. The putative scissile bond (p1-p1') and the p1 residue were predicted based on the conserved features that there are generally 17 amino residues (p17 to p1) between the start of the hinge region of the RCL, and scissile bond [11].

### Guide phylogeny tree

In order to determine relationships among tick serpins, a guide tree of 68 tick serpins sequences, 34 full-length serpins from this study, and 34 other tick serpins that were downloaded from GeneBank, and human α_1_-antitrypsin (out group, AAB59495) as an outlier were used to construct the guide tree using the neighbor joining method. Specifications were set for bootstrap values at 1000 replications, gaps proportionately distributed and correction for distance set to a Poisson distribution. To determine the homologous features that influenced the clustering patterns, sequences of each clade were subjected to multiple sequence alignment analyses.

### Structural based sequence alignment and comparative modeling

To determine secondary structures, randomly selected representative sequences of each cluster in the guide phylogeny tree, serpins S1, 19, 22, 34 and 35 were subjected structural based sequence alignment using the web based STRAP (Structure based Alignment Program) . Subsequently, secondary structures were superimposed on the structurally aligned sequences using the human α_1_-antitrypsin tertiary structure (1HP7, [47]) as template. The aligned sequences were subsequently viewed using the GeneDoc sequence analysis software for windows .

### Expression analyses by RT-PCR

OligodT primed salivary gland (SG) and midgut (MG) cDNA templates of unfed ticks and ticks that were fed for 6, 24, 48, 72 and 96 hrs used in this study were a gift from Dr. Shahid Karim, formerly at the University of Rhode Island, now at University of Mississippi. The harvest of tick tissues and synthesis of cDNA were done as previously described by Kotsyfakis et al., [48]. One μl aliquots of these templates were used in a PCR reaction with specific PCR primers (Tables 1 and 2). PCR products were electrophoresed on a 2% agarose gel containing 1 μg/ml ethidium bromide. The tick actin gene (Table 1) was used for PCR template load control. To determine mRNA abundance, densitograms of amplified PCR bands were determined using the web based ImageJ software . To correct for variations in PCR template concentrations, PCR band densities were normalized using the following formula; Y = V + V (H-X)/X where Y = normalized mRNA density, V = observed Ispin PCR band density in individual tissues (MG and SG), H = highest tick actin PCR band density among tested tissues (carcass in this case 24 hrs MG), X = tick actin density in MG or SG [33].

**Table 1:** Gene specific PCR primers to amplify 16 serpins that have basic residues at their P1 site

Serpin gene	Forward primer	Reverse
S1	CAAAGCTGATACAAGACAAGACCAAG	TTAAAGATTATTGACCTGTCCAGCG

S2	AGTGAAGGAACGAAGAGCTCGAC	TTGTGGAGACATCTGAGATCTTCAGA

S4	AACAGCGATGGGAATGGTTTTC	TCGTTGGGCACAGTTAGGGTC

S6	TACCAAGATGAAAGTTCTCGTGACG	CTTGGCTCAAAGGTGGTTCACTT

S7	ATGAAAGTTATCACTGCGTTCCTGT	CTTGGTTGTCTCAGAGGTGGTTCA

S8	TCAAACCTTGTTTACTTGCCCCA	TCAAACCTTGTTTACTTGCCCCA

S9	TCTTCTCGCCCTACAGCATATCC	TGTTTTGAAGCCTGGCGATTGT

S12	ATGTTCGCTCCGACTGCCCT	GTTAATTTTTCACGTTCTCAAGCCTG

S14	ATGAAGGCTTCAACGGTGTGG	GGTAATAAAAGTGGTCGCACACAATG

S16	GGTGAGTCAAGATGGCAAAATGCC	GTTTACCTGTCCTGCAAACAGCACC

S17	GAGACGAGACAGACCAATTACCTCA	GGATGACTCAGAGGGCGTTGAT

S18	TATCACAACCTCCACGATGTTCG	GTTAATTTCTCACGTTCTCAAGCCTG

S19	ATGTGGTTCCCGGCGCTCCT	CGTCCTCGATGTATTCACAAGTCCAGC

S21	CTGTTCCTCCTGATGGCGGTA	ATTTCTCACGTTCTCAAGCCTGC

S26	ATGCTTGCGGAAAAGGTTTTC	CTGGACTTCTCCCAAAAATAGCG

S27	AAGCTAGATCGACGCCATGAAG	CTAAACCCGCTTCAACATGCTG

S32	GCATCCAGAGCTTCCAAACATG	TCAATGTGATAGGAAGCACTTCGAG

Tick Actin	GGACAGCTACGTGGGCGACGAGG	CGATTTCACGCTCAGCCGTGGTGG

**Table 2:** Gene Specific PCR primers used to amplify transcripts of *I. scapularis *serpins that have non-basic residues their P1 sites

Serpin gene	Forward primer	Reverse primer
S3	ATGAAGTACCTGGTTACGTTCCTC	TTAGAGCTTGTTGACCTGTCCCGC

S5	ATGAGGTCTCTCGCAACGTTCATG	TCAAAGATGATTGACCTGTCCCAC

S10	CGGCCTCAACCTCCTCAGGGAGC	CAGCGATTGATCTACCGCGTCCC

S11	CGACTGCAGCTGCGAGTGCCC	CTGCTCTTTCTTCTGTCACCTC

S13	CTCCGTGGAAGTCGCTATGGTC	GGTGTTGGCAAGTCTGTCCCTG

S15	ATGTCTCCCCGAGGTACCTTTCTC	TTTATGAACCGCACCAATAAAGTG

S20	ATGAAACGCTGCACCCTGGTCGC	TCACACCTCTTGAAGCCTTCCGAG

S22	ATGTACGCTCCGACTGTACTGTTCC	TCAAGCTTGCAACTGGTGCACCTC

S23	CGCCATGGTCTACGCCGGCGCC	CACACGGCCGGTTGCCTTCTCGC

S24	CAGCGAACAGCCGGAATCGAAC	GCCTATTAACGGCGAAGGATGG

S26	ATGCTTGCGGAAAAGGTCTTCTTC	CTTGCCTCTGGTCTTGTTCTTCAC

S28	GTTTGCCGGAAAGGTGTTTCTCG	GTGGCCGAGGGCAGTGGAAAG

S29	ATGATGCTTGCCGAAAAGGTTTTTC	CTAGCCAAGGGCGGGCTTAACGGC

S30	ATGGCTTCCGATTTTGGCGATTC	GATTTGGCGTACAGAGCCAAG

S31	GCCGAACTCCGGGACTCCATG	CACTTCTCCCAAAAATAGCACACG

S33	CTCGAGCTTCCTTTGCAAGCTACCC	GGATGGATCCCATGAAGAGAACAG

S34	ATGGCGATATTACCTGCTCTGAC	GCATGTCGGAGACGAGGTTCCTG

S35	ATGGACGCGGTTTCGAGATTCCTG	CTATAGGTGACGCACCGAACCC

S36	ATGATGTTGGGCCGTCTTGCAGC	TTACAGCTTGTTGACTTGACCAGC

S37	GGAGGTCAATGCCTGGGTCGAG	GCTGCAGATCAGGAACATGAACG

S38	ATGGCTATGGCCTACGCTGGAG	CTAGAGTGCGTTGATATGTCCCAC

S39	ATGGCGTCTGATTTTAGTAATTCTC	TCAGATTTGGGCTACAGAGCCAAT

S40	TCTGAAGGAATTTCTCCAGAAGTTG	TCAAAGCATGTTTACTTGCCCC

S41	ATGAAGACTCTGGCAGCATTCCTG	TTAAGCGGCCGCCTCGGTGCCTTC

S42	CTTTCCTCAAGGTTCAAGTTTGAGAC	CTTTCCTCAAGGTTCAAGTTTGAGAC

S43	GAAACCATGACGAGACGCGGTAAG	GCCGGTTGCCTTCTCGCGGATGTAG

S44	GTCGAGAGCATAGAAAAAATCCAGG	GAGACGGCCGGTAGCCTTCTCGCG

S45	CGCCTCTAGCGAGCGATTTCCTC	GACACCTTCCATTCCGGCGTCGG

## Results

### Discovery of *I. scapularis *serpin genes

**Figure 2:**
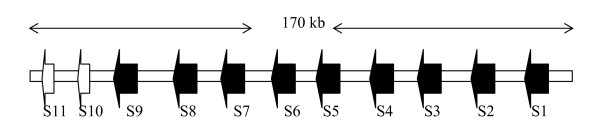
**A cluster of 11 serpin (S) genes (S1-to-11) spanning 170 kb of DNA sequence**. Structurally, nine serpin genes, S1-to-9 have introns, while S10 and 11 are intronless. When scanned against the whole genome shotgun DNA sequence database, encoding these open reading frames of these genes are ABJB010004033 for S1, ABJB010065261 for S2, ABJB010418850, ABJB011135496, ABJB0109755250 and ABJB010321224 for S3, ABJB011011198 for S4, ABJB010283366 for S5, ABJB011103238 and ABJB010459188 for S6, ABJB010459188 for S7, ABJB010459188 for S8 and ABJB010543279 for S9, 10 and 11. Based on genomic DNA sequence, complete open reading frames (ORF) were detected for all serpins except for S11 and S6, which are partial and their ORFs were assembled from EST sequences indicated in Table 3. Open arrow heads (⇐) denote intronless, closed arrow heads (⇒) denote intron-containing genes.

**Figure 3:**
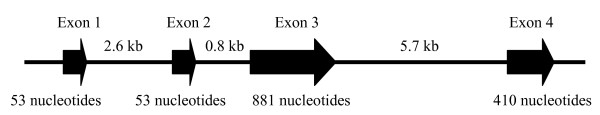
**Typical structure of intron-containing serpin genes**. The cDNA sequence that were assembled from trace archive EST sequences were aligned with supercontig DNA sequences using the Spidey mRNA and genomic DNA alignment software at NCBI [43] and identified the donor-acceptor splice sites (GT-AG). Displayed here is S3 whose cDNA was assembled from trace archive EST and its mate, accession #s 1446657576/1446661330 respectively. The cDNA was mapped onto genomic DNA sequences corresponding to whole genome shotgun sequence (ABJB010418850, ABJB011135496, ABJB0109755250 and ABJB010321224). The first and second exons encode the 5' untranslated region (UTR) and while the third and fourth exon encode the open reading frame, and the 3' UTR.

We successfully used homology search engines, Fasta3 version 3.4 [41], BLASTX and BLASTN to scan supercontigs of the *I. scapularis *genome and annotated 45 serpin genes in the *I. scapularis *genome (Table 3). Of the 45 genes, serpins (S) 1 to 11 occur in a cluster spanning 170 kb of DNA sequence (figure 2) and the rest occur singly or in pairs. When scanned against the *I. scapularis *trace archive cDNA database, 72% (32/45) of the serpin coding regions showed matches in the EST database with e-values of zero (probability that this observation was not by chance) (Table 3). The delineation of introns and exons boundaries based on Spidey [43] and Genome Scan [42] programs revealed that, the 45 serpin genes are structurally segregated into 32 intronless and 13 intron-containing genes. The intron-containing genes, have four exons with the first and second exon coding for the 5' untranslated region (UTR) and the third and fourth exons coding for the open reading frame and the 3' UTR (figure 3). Of the 13 intron-containing genes, nine belong to the cluster of 11 genes shown in figure 2.

**Table 3:** Summary, characterization of serpin inferred amino acid sequences

**Serpin # ID**	**Signal peptide**	**Putative Function**	**Trace archive EST/mate Accession #s**	**Whole genome shotgun accession #s**	**Best match (% identity)**
1	+	Inhibitory	1731419061/1731424162	ABJB010004033	IriS-4, DQ915844 (56)

2	+	Inhibitory	1446657576/1446661330	ABJB010418850 and ABJB011135496	IriS-4, DQ915844 51)

3	+	Inhibitory	1446665747/1446661466	ABJB010065261	IriS-4, DQ915844 (56)

4	+	Inhibitory	1446883315/1446875792	ABJB011011198	IriS-4, DQ915844 (57)

5	+	Inhibitory	1446915260/1446919205	ABJB010283366	IscAAM93649 (97)^REF1^

6	+	Inhibitory	1446658181/1446658460	ABJB011103238	IscAAV80788 (73)

7	+	Inhibitory	1446654646/1446653845	ABJB011103238 and ABJB010459188	IscAAV80788 (97) ^REF1^

8	+	Inhibitory	NEF	ABJB010459188	IscAAV80788 (73)

9	+	Inhibitory	1798691964/1798683282	ABJB010543279	IscAAV80788 (73)

10	P	Inhibitory	NEF	ABJB010543279	Iri-1 DQ915842 (95)

11	+	Inhibitory	NEF	ABJB010543279	Lospin17 ^REF2^(46)

12	+	Inhibitory	1798658594/no mate	ABJB010080534	Lospin17 ^REF2^(29)

13	+	Non-Inhibitory	1798680529/1798669906	ABJB010568406	HloS-2, AB162827 (50)

14	+	Inhibitory	1446647997/1446656254	ABJB010572532	Lospin17 ^REF2^(38)

15	+	Inhibitory	NEF	ABJB010589966	Lospin17 ^REF2^(48)

16	+	Inhibitory	NEF	ABJB010189666	Lospin17 ^REF2^(44)

17	+	Inhibitory	1446918763/1446915174	ABJB010803180	IriS-1 DQ915842 (30)

18	+	Inhibitory	1446686436/1446684945	ABJB010296835	IriS-8 DQ915845 (98)

19	+	Inhibitory	1446935509/1446936853	ABJB010938637 and ABJB010859665	HloS-2, AB162827 (50)

20	+	Inhibitory	1446937497/1446940930	ABJB010863599	Lospin10 ^REF2^(34)

21	-	Inhibitory	799333999/1799375613	ABJB011069597 and ABJB010801481	Lospin17 ^REF2^(52)

22	+	Inhibitory	1798749904/1798771804	ABJB010325534 and ABJB010620410	HloS-2, AB162827 (51)

23	+	Inhibitory	1702098781/1702102057	ABJB010111671 and ABJB010862034	HloS-2, AB162827 (50)

24	+	Inhibitory	NEF	ABJB010229324	Lospin4 ^REF2^(46)

25	+	NR	NEF	ABJB010327382	Lospin17 ^REF2^(41)

26	+	Inhibitory	1702098781/1702098781	ABJB011108013	HloS-2, AB162827 (44)

27	+	Inhibitory	1446877265/1446875646	ABJB010216105	HloS-2, AB162827 (48)

28	+	NR	NEF	ABJB011030283	Lospin4 ^REF2^(34

29	+	Inhibitory	1702098781/1702102057	ABJB010216676	Lospin17 ^REF2^(49)

30	-	Inhibitory	1798755892/1798766236	ABJB011013454	Lospin12 ^REF2^(43)

31	P	Inhibitory	NEF	ABJB010583840	Lospin17 ^REF2^(47)

32	-	Inhibitory	1799314980/1799282524	ABJB010837586	Lospin7 ^REF2^(61)

33	P	Inhibitory	1446912351/1446909212	ABJB010588517	Lospin17 ^REF2^(44)

34	+	Inhibitory	NEF	ABJB010122781	Iris ^REF2^(93)

35	-	Non-inhibitory	1701896038/1701906143	ABJB010889434	IriS-2, DQ915843 (46)

36	+	Inhibitory	1446674847/1446673722	ABJB010262605	Iris ^REF3^(93)

37	-	Inhibitory	1446912351/1446909212	ABJB010112590	IriS-2, DQ915843 (60)

38	-	Inhibitory	1446875683/1446883661	ABJB011132035	Iris ^REF3^(60)

39	P	Inhibitory	1799178683/1799156584	ABJB010146202	Iris ^REF3^(55)

40	P	Inhibitory	NEF	ABJB011073580	Lospin17 ^REF2^(39)

41	+	Inhibitory	1799307827/1799285191	ABJB010040374	IriS-2, DQ915843 (93)

42	+	Inhibitory	1798653329/1798670720	ABJB010592280	IscAAV80788 (66)

43	P	Inhibitory	NEF	ABJB010519540	IriS-1 DQ915842 (61)

44	P	Inhibitory	1701931459/1701895980	ABJB010496686	RapS-3 ^REF4^(44)

45	P	Inhibitory	NEF	ABJB010314107	Lospin7 ^REF2^(41)

^REF1^[35], ^REF2^[33], ^REF3^[34], ^REF4 [^17], NR, denotes partial in the carboxy-terminal region and no reactive center loop found, thus functionality not determined. Plus sign (+) denotes signal peptide is present, P = partial at the amino terminus region and presence of SP not determined. NEF denotes, not EST found. ^2^Because of space these two (ABJB0109755250 and ABJB010321224) additional accession numbers not listed in table above

As summarized in table three, all *I. scapularis *serpin sequences showed best matches exclusively to other tick serpin sequences. Except for S5, 9, 17 and 40 that respectively showed 96, 97, 99, 96 and 95% amino acid identity to *I. ricinus *serpin (Irs) 4 (accession [acc] # DQ915844), Irs1 (acc# DQ915842), Irs8 (acc# DQ915845), the *I. ricinus *immunosuppressant serpin (AJ269658, [34]) and Irs2 (acs# DQ915843), the identity levels between serpins in this study and other tick serpins ranged between 34 to 57% (Table 3). Two of the serpins genes in this study, S5 and 7 are 99 and 98% identical to previously annotated *I. scapularis *serpins, AAM93649 and AAV80788 respectively [35] (Table 3). At the time of preparing this manuscript, a preliminary annotation of the *I. scapularis *genome (version 0.5) was released . When scanned against this database, 88% (40/45) of the serpin sequences produced matches with e-values of zero. The reader is notified here that because neither supercontig accession numbers nor the preliminary gene annotations are referenced in GeneBank, whole genome shotgun sequence and/or trace archive accession numbers are given in table three to provide the source for primary sequence data that was used in this study.

### Inferred amino acid sequence analyses

**Figure 4:**
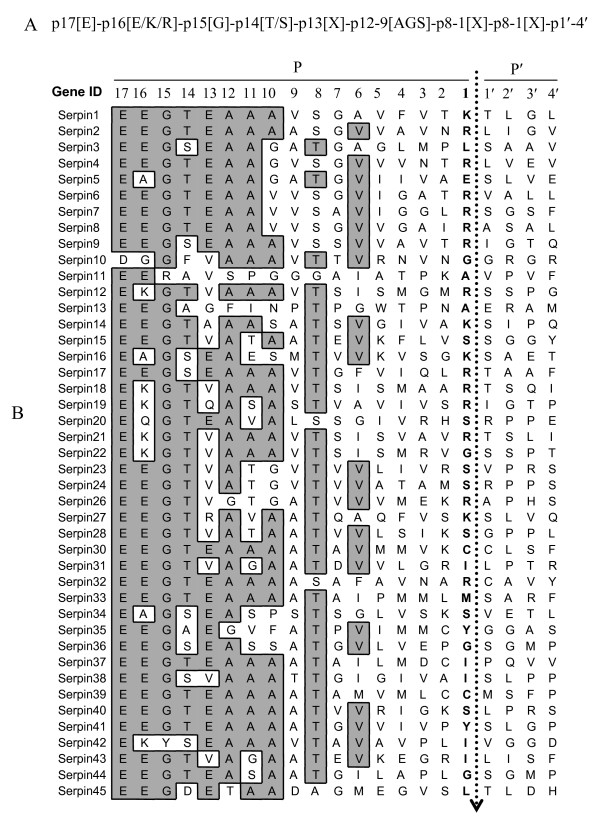
**Predicted *I. scapularis *serpin reactive center loops (RCL) (B)**. RCLs were determined based on the eight, residue pattern (A) that characterizes inhibitory serpins [45]. The residues in the RCL are numbered according to the standard nomenclature developed by Schechter and Berger [46], where residues on the amino-terminal side of the scissile (P1-P1') are not primed and those on the carboxy terminal side primed. Assuming that there are 17 residues between the base of RCL hinge and the scissile bond [45], boldfaced residues are predicted P1 residues with the broken line arrowhead indicating the position of the scissile bond.

A typical full-length serpin molecule may range between 350-to-450 amino acid residues long [11,14]. On this basis and in addition to possessing in frame start and stop codons, 35 of the 45 inferred serpin sequences were determined to be full-length (Table 3). As indicated in table three, eight of the ten partial sequences are truncated at their amino terminus, while the remaining two sequences are truncated at their c-terminal. At their c-terminus, serpins are characterized by a flexible reactive center loop (RCL), the primary determinant of functionality (inhibitory or non-inhibitory) that acts as a pseudo-substrate for the target proteinase [11,42]. Based on consensus amino acid residues [11,14,45], RCLs have been detected in all sequences (figure 4), except for serpin 25 and 28 that are truncated at their c-terminus (Table 3). On the basis that inhibitory serpins have more than 50% of "A" between p12-9 and that charged amino acid residues at p14 or "P" residues at p12 or p10 in the RCL, causes loss of inhibitory function [11], 41 of the 43 serpins that have a RCL are predicted to have inhibitor functions (figure 4). In the RCL the P1 residue determines substrate specificity [11,45]. By consensus there are generally 17 amino acid residues (p17 to p1) between the start of the hinge region of the RCL, and scissile bond [p1-p1'] [11,49]. On this basis, visual inspection of putative RCL amino acid sequences revealed a diversity of 11 different residues K, R, L, G, A, S, E, C, Y, I and M at the P1 sites (figure 3). As shown in figure 2, 16 of the 43 serpin, sequences that have RCLs are predicted to possess basic charged amino acid residues, "R" or "K" at their P1 site (figure 4). Of the 16 serpin sequences, seven belong to the cluster of 11 serpin genes that are shown in figure 1. It is also interesting to note that eight other serpin genes possess basic residues at their P2 site.

### Amino acid motifs scan analyses

Motif scan analyses on the ScanProsite server [50] revealed that the serpin signature motif pattern PS00284 ([LIVMFY]-G- [LIVMFYAC]- [DNQ]- [RKHOS]- [PST]-F- [LIVMFY]- [LIVMFYC]-X- [LIVMFAH] was present in all sequences (results not shown). On the SignalP3.0 server , 89% (31/35) of the 35 full-length serpin sequences were predicted to have leader sequences (Table 3). Other important motifs include the putative N-glycosylation (NX [S/T]) sites in 38 of the 45 serpin sequences. Except for S11, which was predicted to have eight putative N-glycosylation sites, the rest have between one to three sites. We also found the cell attachment motif "RGD" in four sequences, S1, 7,17 and 42.

### Guide phylogeny analysis

**Figure 5:**
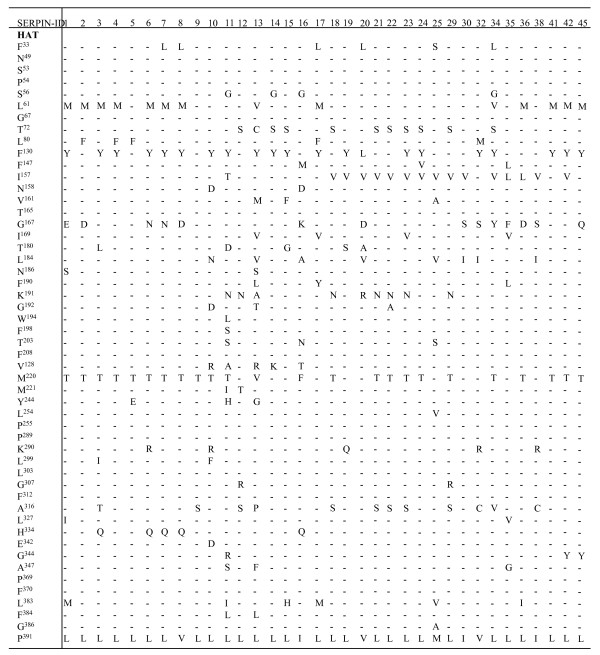
**Conservation of core amino acid residues adopted from **[51]. 35 full-length *I. scapularis *serpin sequences were aligned with the human, α_1_-antitrypsin (α_1_-HAT, accession # AAB59495) using structural based sequence alignment program (STRAP, . The aligned sequences were visually inspected for conservation of core amino acid residues. The minus sign (-) denotes conservation of the specific amino acid residues. Replaced residues are indicated.

In order to determine the relationship among tick serpins, we used the neighbor joining method to generate a phylogeny guide tree of 68 tick serpin sequences. From the α_1_-antitrypsin outlier, 68 tick serpin sequences segregated into five main clusters, A-E supported by 93, 90, 96, 88 and 93% bootstrap values (figure 5). Except for Bmserp1, all other sequences in main branch "A" have no signal peptides. The remaining sequences, all of which have signal peptides are segregated into branches "B-E".

### *I. scapularis *serpins posses key residues that characterize the secondary structure the serpin superfamily

**Figure 6:**
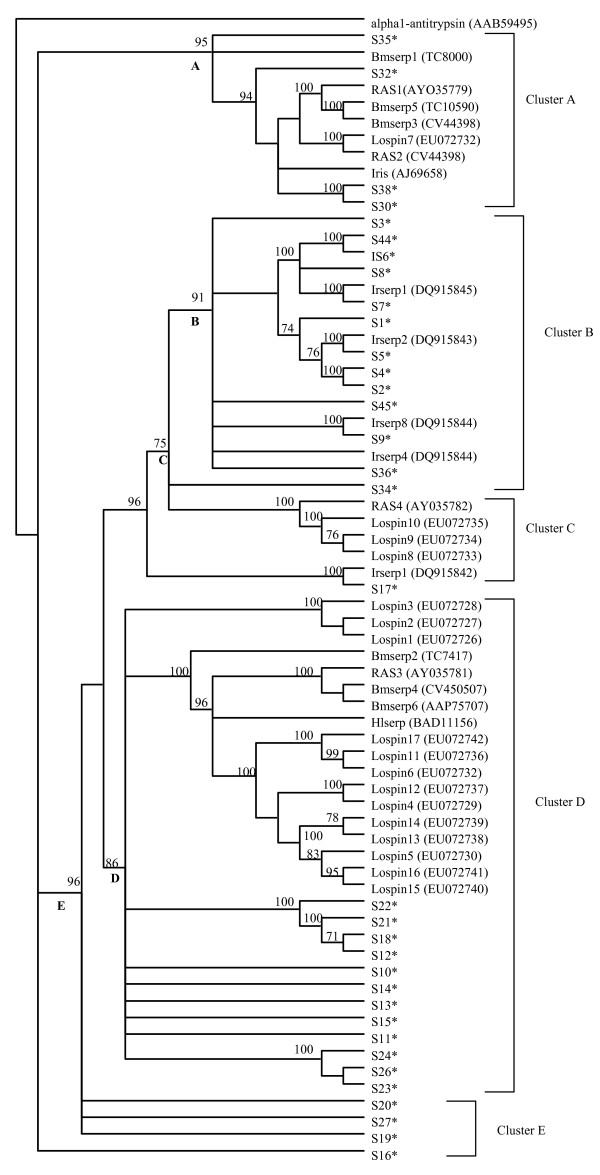
**Neighbor joining guide phylogeny tree**. Deduced amino acid sequences of 35 full-length serpins were aligned with 17 serpins from *A. americanum *[33], *R. appendiculatus *serpin (Ras) -1-to-4 [17], *H. longicornis *(Hl) serpin, *I. ricinus *(Irserp) serpin 1, 2, 4, 8 and Iris = immunosuppressive *Ixodes *serpin, *I. scapularis *(Is) serpin and *B. microplus *(Bm) serpin 1-to-6. Note that, except for Bmserpin-5, which, is annotated in GenBank, the other Bm serpins were obtained as ESTs from the TIGR database and translated in Mulenga et al., [33]. Cluster labels "A-to-E", represents branching from the outlier, the serpin superfamily archetype, α_1_-antitrypsin. Accession numbers of whole genome shotgun genomic DNA sequences corresponding to supercontig sequences used in the annotation of *I. scapularis *serpins (marked with an asterisk sign) are given in Table 3.

Irving et al., [51] performed a sequence alignment of 219 serpin sequences and identified 51 core amino acid residues that underlie the functionality of inhibitory serpins. Here we adopted this analysis by aligning the 35 full serpin sequence with the human α_1_-antitrypsin (accession # AAB59495) and found that the 51 core residues that underpin the efficient functioning of an inhibitory serpin [51] are 67–96% (34–49/51) conserved in *I. scapularis *serpins (figure 6). The inhibitory mechanism of the serpins relies on their structural flexibility, which is controlled by the shutter region [11,52]. Two amino acid motifs S^53^-P^54^-X^55^-S^56 ^and I^157^-N^158^-X^159^-X^160^-V^161 ^(numbering is based on the serpin human α_1_-antitrypsin) that underpin the functioning of the shutter region [11,52] are 100% conserved in 20 of the 34 *I. scapularis *serpin sequences (figure 6).

**Figure 7:**
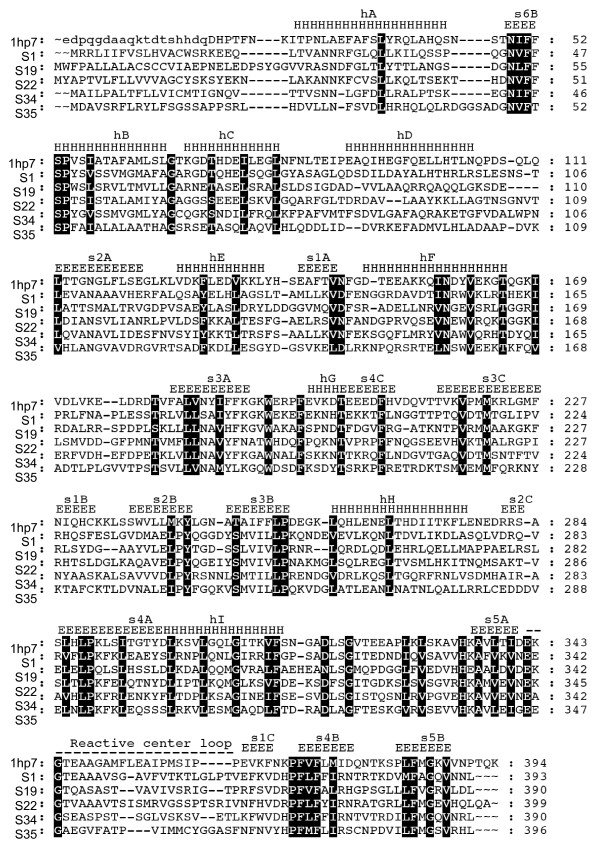
**Structural-based multiple sequence alignment**. Using human α1-antitrypsin (1hbp7) as modeling template, five serpin sequences (S1, S19, S22, S34 and S35) representing each of the five main branches in figure 4 were aligned using the structural based sequence alignment program (STRAP, ). Secondary structures assigned based on α_1_-antitrypsin tertiary structure (PDB ID, 1hbp7), are labeled as "H" for α-helix and "E" for beta-strand. Helices are labeled from "hA" to "hI", β-strands that constitutes β-sheet A-to-C are labeled as "sA", "sB" and "sC" respectively. The serpin superfamily core residues [52] are shaded in black. The reactive center loop domain is noted.

Given the high conservation of core residues that underpin the functionality of serpins, we thought to determine whether or not the serpin common fold of 7–9 α-helices and three β-sheets (A-C) and the RCL [12] were also conserved in the deduced serpin sequences. As shown in figure 6, structural based sequence alignment of representative sequences (S1, 19, 22, 34 and 35) from each of the five main branches shown in figure 4, revealed that the serpin secondary structure is conserved in *I. scapularis *serpins (figure 7).

### Transcriptional analyses

**Figure 8:**
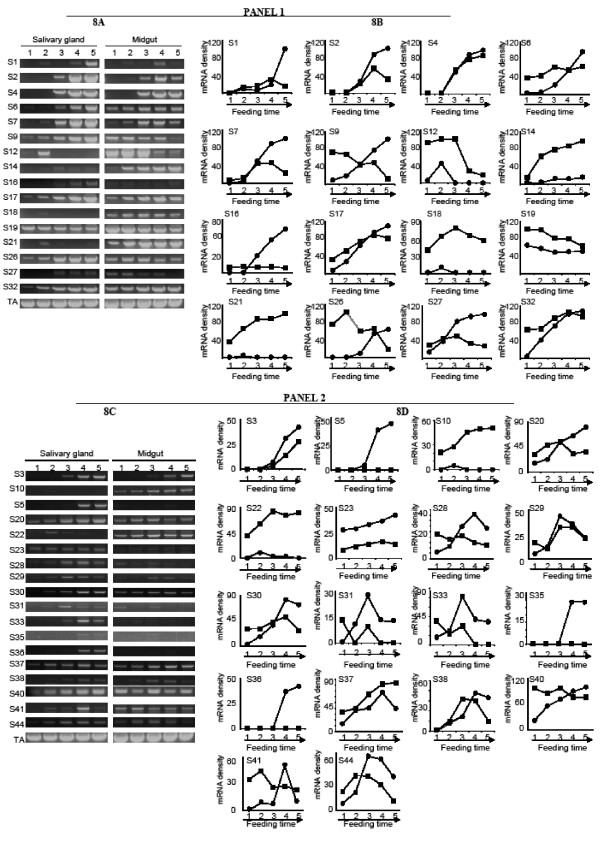
**Transcription profiles (8A and 8C) and normalized PCR band densities representing relative mRNA abundance (8B and 8D)**. cDNA synthesized from total RNA of salivary glands (SG) and midgut (MG) of unfed and ticks that were partially fed for 6 to 96 hrs was subjected to semi-quantitative RT-PCR using gene specific primers shown in tables 1 and 2. PCR band densities were determined using the ImageJ software . Determined densities were normalized using the following formula, Y = V + V(H-X)/X where Y = normalized mRNA density, V = observed Ispin PCR band density in individual tissues (MG and SG), H = highest actin PCR band density, X = tissue (MG and SG) actin band density.

To begin determining the role of serpins in this study in tick feeding and physiology, semi-quantitative RT-PCR was used to establish temporal and spatial expression patterns in salivary glands (SG) and midguts (MG) of unfed and ticks that were partially fed for 6, 24, 48, 72 and 96 hrs (figure 8). Of the 45 serpin sequences, 40 are expressed in SG and MG; 34 being strongly expressed (figures 8A and 8B) and five weakly expressed (not shown). For clarity and limitations on space, we have presented our RT-PCR data in two panels. Panel one (figures 8A and 8B) show expression patterns of serpin sequences that possesses basic residues (R/K) at their P1 sites, while expression patterns of the rest of the genes are displayed in panel two (figures 8C and 8D). On the basis of visual intensity of PCR bands (figure 8A and 8C) and analysis of normalized PCR band densities (figure 8B and 8D), expression patterns of the 35 genes can be segregated into four profiles. In the first pattern, six genes (S1, 2, 4 and 7 in panel one, S3, 28, 38 and 44 in panel two) whose transcript increased with tick feeding were expressed from six or 24 hrs of tick feeding. The next set of genes, are constitutively expressed in both MG and SG with their transcript abundance either increasing (S17 and 32 in panel one, 23 and 37 in panel two) or decreasing (S19 in panel two) as ticks continued to feed. The third group of genes S6, 9, 12, 16, 21, 26 and 27 in panel one and S20, 25 and 40 in panel two displayed a dichotomous expression pattern. In the case of S6, it is expressed from 24 hrs in SG but constitutively expressed in MG with its transcript abundance increasing in both organs as ticks continue to feed. In the case of serpin S16 and 26, which are induced from 24 hrs in SG and their transcript abundance increasing thereafter, are constitutively expressed in MG with transcript abundance decreasing as ticks continue to feed. For S9 and 27 in panel one, S20, 25, 30, 33, 40 and 41 in panel two, they are constitutively expressed in both organs, but their transcript abundance increases in SG and decreases in MG as ticks continue to feed. The fourth profile consist of genes that are predominantly or specifically expressed in SG (S1, 5, 16, 31, 35 and 36) and MG (S10, 14, 18, 21 and 22) with transcript abundance decreasing for the former and increasing for the later as ticks continued to feed.

## Discussion and conclusion

Analysis of genome sequence data has led to the discovery of large families of serpins in multicellular organisms, including 36 in humans, nine in *Caenorhabditis elegans *[16], 29 in *Drosophila *[26] and plants [53]. On the Merops database, 17 and 18 serpin sequences are listed for *Aedes aegypti *and *Anopheles gambiae*. In ticks, documentation of multiple serpin encoding cDNAs has provided indirect evidence suggesting that ticks do encode large serpin families. For instance, we recently described 17 serpin cDNAs that are expressed by *A. americanum *during feeding [33]. Here we describe the annotation and characterization of 45 serpin genes in the *I. scapularis *genome. The observed high amino acid sequence identity between *I. scapularis *and *I. ricinus *serpins was not surprising as these two ticks belong to the same genus. It is important to point out that eight of the 45 annotated were not represented in the preliminary peptide build at VectorBase. This finding may raise the prospect of error in annotations reported here. Interestingly, this possibility is ruled out, as ESTs of six of the eight serpins in this study (S1, 13, 23, 24, 33 and 39) were present in the trace archive database, while coding regions of serpins S26 and 32 were amplified from unfed and partially fed ticks (see figure 7).

The adoption of the consensus serpin secondary structures [11,12] and the high conservation of the core amino acid residues [51] that underpin the structure and functionality of serpins strongly suggested that, *I. scapularis *serpins are functional. The majority of known serpins function as inhibitors of serine proteinases and hence the name [54]. However others with activity against cysteine proteinases and those with no inhibitor functions have also been described [12]. Although on the basis of sequence analysis [11], we are able to distinguish between inhibitory and non-inhibitory serpins, available data in this study is insufficient to specify their preferred proteinase substrates. Putative RCLs and scissile bonds of serpins in this study were predicted based on consensus that there are 17 amino acid residues between the start of the RCL hinge region and the scissile bond [11,45,51]. As some of the characterized serpins such as α_2_-antiplasmin [11] or serpin1k from *Manducca sexta *[55], utilize shorter RCLs, we are interpreting our predictions of RCLs and scissile bonds with caution.

Our finding that 82% of the full-length serpins in this study have signal peptides is consistent with observations in humans where the majority of known serpins exist in the extracellular form [14]. Findings in this study are not unique, in that similar results were reported in *A. americanum *where 13 of the 17 putatively inhibitory serpins in *A. americanum *were predicted to be extracellular [33]. From the perspective of finding target antigens for vaccine development, it is encouraging to note that the majority of *I. scapularis *serpins are putatively extracellular as they will be accessible to host immune response factors. Predictions based on sequence analysis, may not be consistent with the situation *in vivo*. However it is interesting to note that the four serpin sequences (S30, 32, 35 and 38) that were predicted to be intracellular sequences, based on lack of a signal peptide also posses "C" residues in the exposed regions of their RCL, a feature that has been observed in many intracellular serpins [14].

The use of alternatively spliced RCLs appears to be a wide spread strategy in insects to diversify the range of target proteinases that can be controlled by a single serpin gene [56-58]. A classic observation of this phenomenon is the serpin gene-1 of the tobacco hornworm, *M. sexta*, which has 12 different alternatively, spliced RCLs [56]. Effectively this gives rise to 12 serpins regulating 12 different proteolytic pathways. An interesting structural feature among the 12 variants of *M. sexta *serpin gene 1 is that the first 336 amino acids are exactly identical with difference restricted to the RCL [56]. The identity patterns among *I. scapularis *serpins sequences of where, stretches of identical and variable domains were spread across the entire sequence suggest that the diversity among serpins in this study may have arisen by duplication other than alternative splicing of RCL encoding exons.

From the perspective of understanding how the tick manipulates the mammalian host's defense against tick feeding, the finding of 18 serpins with basic residues at their P1 sites was exciting. In humans, this feature is associated with key serpins such as α_1_-antichymotrypsin, α_1_-antiplasmin, antithrombin III, protein C inhibitor and C1 inhibitor [11], which regulate important pathways such as inflammation, blood coagulation and complement activation. As these pathways are thought to represent the mammalian host's defense against tick feeding [59,60], it is tempting to imagine that ticks could utilize some of these serpins to manipulate host defense to facilitate tick feeding and disease transmission. It is also possible that these serpins may not be directly be involved in facilitation of feeding. However, like their mammalian counterparts, they may be involved in regulation of important pathways in the tick, which if disrupted can affect the capacity of ticks as vectors.

Although the biological significance of gene expression data will be strengthened if correlated with protein production, our RT-PCR data provide some useful insights on probable biological roles of serpin genes in this study in the physiology of *I. scapularis*. Speculatively *I. scapularis *genes that were induced or up regulated after ticks had penetrated their host skin may signal their involvement in facilitation of blood meal up take. This is particularly true for the 11 genes that were induced in both SG and MG (S1, 2, 3, 4 and 7) or SG alone (S5, 6, 16, 26, 35 and 36) in ticks that were fed for 6–24 hrs. This period corresponds to the preparatory feeding phase when tick attaches onto host skin and creates its feeding lesion [2]. During this period the tick must overcome inflammation and blood coagulation for it to successfully start the feeding process. Similarly, the group of serpin genes (S17, 23, 25, 32, 37, 38 and 40) that were constitutively expressed but their transcript abundance increased with tick feeding may also play a role in facilitating blood meal up take. For those genes that were constitutively expressed, S9, 12 and 27 in the MG, and S19 in both SG and MG, but were progressively down regulated as ticks continued to feed, could be involved in regulating physiological processes at the front end of tick feeding process. The expression of S10, 14, 18, 21 and 22, specifically or predominantly in the MG is interesting as it signals the potential role for these genes in facilitation of not only blood meal processing, but also in the crossing of the gut barrier by pathogens. From the perspective of our long-term interest to find tick proteins that are used by ticks to evade host immunity, it was exciting to note that some serpins were specifically expressed in SG. It will be exciting to investigate whether or not any of these genes are injected into the host during tick feeding. It is possible that the genes analyzed here could be expressed in multiple tick organs besides the SG and MG. However, from the perspective of our long-term interests to understand molecular mechanisms that underlie tick-host interactions, our analysis here is biased to biological functions of serpins at the SG and MG levels. The SG is critical for feeding and disease transmission while the MG is important for blood meal processing and the passage of pathogens from the blood meal into the tick hemolymph [2]. Our future questions will thus address the role of the serpins in facilitation of tick feeding and blood meal processing.

Most known serpins are glycosylated [12,16] and thus it is not surprising that 40 of the 43 serpin sequences that were tested are predicted to possess putative N-glycosyslation sites. As pool feeders, ticks accomplish feeding by lacerating small blood vessels and then sucking blood from the hematoma that forms in the feeding site [2]. In order to complete feeding, ticks must prevent host blood from coagulating to ensure continued blood flow into the hematoma for the entire tick feeding period, which may last for over the 10–14 and 4–7 day feeding periods for adults and immature ticks respectively [2]. From the perspective of solving the paradox of how the tick interferes with the coagulation cascade of its mammalian host, it was interesting to note that ~9% (4/45) of the sequences contain the RGD motif. Previous studies have shown that tick proteins containing the RGD motif such as variabilin [61] and savignygrin [62] were potent inhibitors of platelet aggregation. Platelet aggregation is critical to stopping bleeding of injured small blood vessels such as occurs at the tick bite [59]. Thus, if functional, the RGD motif containing serpins could represent important molecular targets aimed at countering the ability of ticks to suppress the mammalian's host's blood clotting system. In addition to the anti-platelet aggregation function, RGD motif containing proteins are also involved in regulating cell-cell interactions in mammals [63], immunity in arthropods [64], and plants [65]. From the foregoing, it is clear that the RGD motif containing serpins could also be involved in regulation of multiple other functions in the tick, besides platelet function at the tick feeding site. It is interesting to note that our RT-PCR data suggested that the expression of three (S1, 7 and 16)) of the four serpin RGD motif-containing genes was responsive to tick feeding activity (see figure 8).

When compared to the 3100 Mbp human genome that encodes at least 36 serpin genes [16] the 45 serpin genes identified in *I. scapularis*, which has a 2100 Mbp genome is considerably high. While the biological significance of the high number of serpin genes in the *I. scapularis *biology cannot be ascertained at present, we speculate that this may signal the significance of serpins in tick physiology. In light of lack of genome sequence data of many tick species, the sizes of tick serpin families will remain unknown. Ticks are diverse, both in terms of their biology [2] and their genome sizes [66-68]. Thus it is likely that the sizes of serpin families in ticks are going to vary. However, this study provides some insight on the probable sizes of serpin families in ticks.

## Authors' contributions

AM was responsible for conception, design, data interpretation, discussion and presentation of the work. RK equally participated in the conception, was responsible for data mining and assembly and performed part of the transcription analysis. KCC was responsible for completing all transcription analysis. All authors participated in the write up of the manuscript.
